# Supplementary material for: Relaxation and revival of quasiparticles injected in an interacting quantum Hall liquid
Source: Nat Commun. 2020 May 15;11:2426. doi: 10.1038/s41467-020-16331-4 (PMC7229030; doi:10.1038/s41467-020-16331-4)
Supplement: Supplementary file 1 — Supplementary Information [file 41467_2020_16331_MOESM1_ESM.pdf]

Supplementary Information for
Relaxation and revival of quasiparticles injected in an interacting quantum Hall liquid

R. H. Rodriguez,[1] F. D. Parmentier,[1] D. Ferraro,[2,3] P. Roulleau,[1] U. Gennser,[4] A. Cavanna,[4] M. Sassetti,[2,3] F. Portier,[1] D. Mailly,[4] and P. Roche[1]

[1] *Université Paris-Saclay, CEA, CNRS, SPEC, 91191, Gif-sur-Yvette, France*
[2] *Dipartimento di Fisica, Università di Genova, Via Dodecaneso 33, 16146, Genova, Italy*
[3] *SPIN-CNR, Via Dodecaneso 33, 16146, Genova, Italy*
[4] *Centre de Nanosciences et de Nanotechnologies (C2N), CNRS, Université Paris-Sud, Université Paris-Saclay, 91120 Palaiseau, France*

(Dated: April 17, 2020)

SUPPLEMENTARY NOTE 1: MEASURED SAMPLES

Devices and datasets

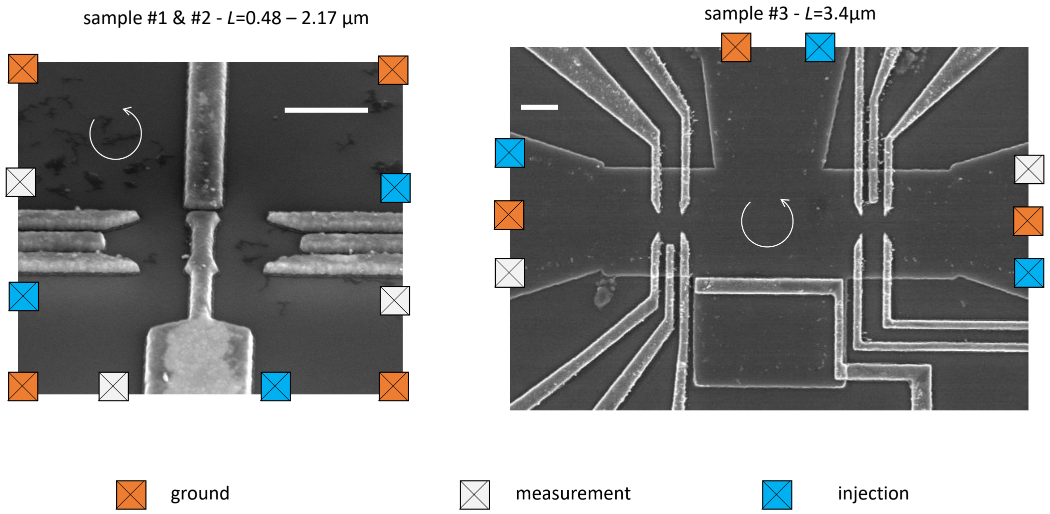

Supplementary Figure 1 | Samples. Scanning electron micrographs of typical sample geometries (left: samples #1 and #2, $L = 0.48 - 2.17$ μm; right: sample #3, $L = 3.4$ μm). The circular arrow indicate the chirality of the edge channels. The white scale bars correspond to 500 nm. The ohmic contacts are symbolized by the crossed squares, the color of which indicate the contact's role in the measurement circuit (orange: cold ground, grey: ac and dc current feed, blue: measurement).

All data discussed in the main text were measured in three samples (#1, #2 and #3). Typical geometries, as well as ohmic contact layout and configuration, are shown in Supplementary Fig. 1. A complete set of curves (referred to in the following as a spectrum), as shown in the figures of the main text is obtained using a unique pair of resonances of the QDs. Supplementary table 1 summarizes the resonances implemented in the emitter (QD1) and the detector (QD2) for each spectrum (S1 to S8), the corresponding figure of the main text, the propagation length L, the respective transmission $T_{1,2}$ and width $\Gamma_{1,2}$ of emitter and detector resonances (see below), the characteristic decay energy E_d, and the average width (FWHM) of the detected QP peak.

Sample	Data	Res-QD1	Res-QD2	Fig.	L<br>μm	T_1	Γ_1<br>μeV	T_2	Γ_2<br>μeV	FWHM<br>μeV
#1	S1 - 480 nm res. A	1A	2A	2, 3a, 4, 6	0.48	0.44	12.8	0.3	5.9	19
#1	S2 - 480 nm res. B	1B	2B	4, 6	0.48	0.60	18.7	0.50	11.25	30
#2	S3 - 750 nm cooldwn 1	1C	2C	2, 3b, 4	0.75	0.54	16.3	0.34	9.6	29
#2	S4 - 750 nm cooldwn 2	1C	2C	2, 3b, 4	0.75	0.50	15.0	0.50	10.0	22
#2	S5 - 750 nm short	1C	2C	5d	0.75	0.9	15.0	0.19	9.8	29
#2	S6 - 750 nm sep.	1D	2D	5e	0.75*	0.40	11.8	0.55	4.9	18
#2	S7 - 750 nm long	1E	2E	5f, 6	2.17	0.43	17.0	0.20	27.0	–
#3	S8 - 3400 nm	1F	2F	6	3.4	0.25	19.75	0.43	8.0	–

Supplementary Table 1 | Measured samples and the corresponding data discussed in the main text. The column Res-QD1 (Res-QD2) refers to the resonance in the emitter (detector) QD used to measure each spectrum. Spectrum S6 corresponds to the $L = 750$ nm dataset with separated ECs.

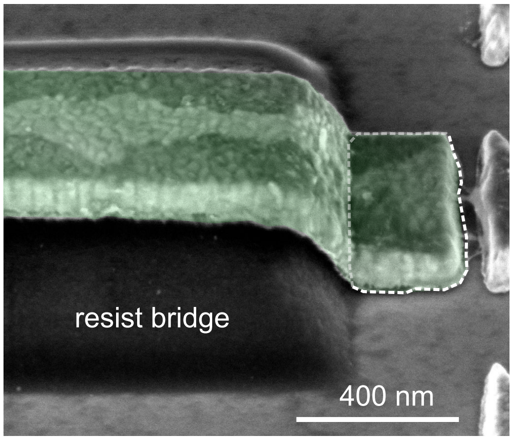

Supplementary Figure 2 | **Length gate.** Scanning electron microscope side view of the side gate, showing the insulating SU-8 resist bridge separating the gate from the surface of the sample. The imprint of the gate on the 2DEG is highlighted by the white dashed line.

Length gate

A side view of the length gate is shown in Supplementary Fig. 2, displaying the 200 nm-thick SU-8 resist layer separating the left part of the length gate from the surface of the sample, greatly weakening it electrostatic influence on the 2DEG. As a result, when applying a voltage to that gate, only the $\approx 300x500$ nm region of the 2DEG at the end of the gate (circled with a white dashed line in Supplementary Fig. 2) sees a change in carrier density. Without this resist bridge, the two quantum dots would become electrically isolated by the depleted region extending all across the 2DEG.

Supplementary Fig. 3 depicts how the short and long path lengths are extracted from scanning electron micrographs for the 750 nm device. The same method is used to extract the lengths for the 480 nm device.

SUPPLEMENTARY NOTE 2: QUANTUM DOTS CALIBRATION

The resonances used in the emitter ($i = 1$) and detector ($i = 2$) QDs were characterized by measuring the transconductance $\partial I_i/\partial V_{\mathrm{Pi}}$ as a function of the respective bias voltage (V_D for $i = 1$ and V_S for $i = 2$) and the plunger gate voltage V_{Pi}. It is in particular crucial to work with resonances devoid of excited states, so as to be sure that quasi-particles are emitted at a single well defined energy [1]. These usually are easily identified when measuring Coulomb diamonds plots. The color plot in Supplementary Fig. 4 shows a typical measurement in the sequential tunneling

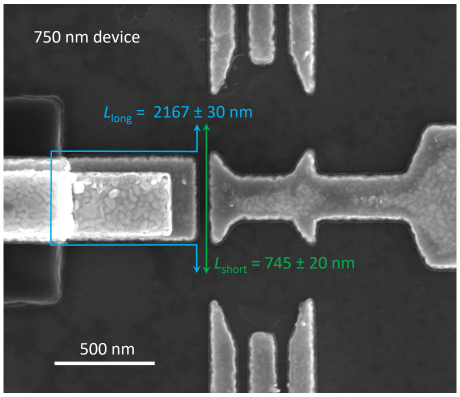

Supplementary Figure 3 | Estimation of the propagation lengths on the 750 nm sample. Scanning electron microscope view of the sample, with the green arrow depicting the short path ($L = 750$ nm) and the blue arrow depicting the long path ($L = 2.17$ μm) circumventing the length gate.

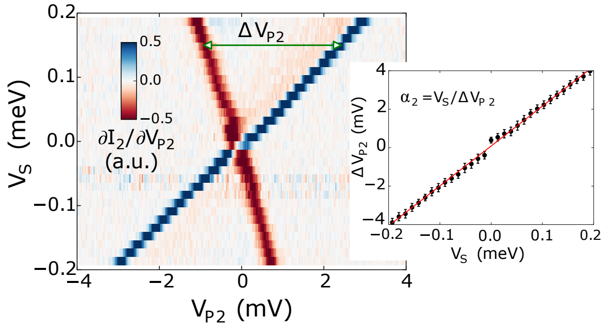

Supplementary Figure 4 | QD calibration. The color map is a typical transconductance measurement of a QD in the Coulomb blockade regime. The inset is the linearisation from which we obtain the lever arm α.

regime. The red and blue oblique lines, separated by ΔV_{Pi}, define the boundaries of the Coulomb blockade regime. A linear fit of ΔV_{Pi} as a function of the bias voltage allows us to extract the lever arm α_i, as shown in the plot at the right hand side. Supplementary table 2 summarizes the lever arm obtained from the calibration of each of the resonances used in this work. The transmission and widths of the resonances (see Supplementary Table 1) are extracted from the differential conductance $\partial I_i/\partial V_{\mathrm{D,S}}$ at zero bias voltage. In our experiment, we have tried to work, for both QDs, with very narrow resonances (with widths ideally smaller than the temperature) so as to maximize the energy resolution, and with close to unity transmission (corresponding to symmetric barriers), so as to maximize the signal-to-noise ratio. In practice, this turns out to be quite challenging, especially when dealing with quantum dots sharing a common depletion gate. The data shown in the main text and in the supplementary information correspond to the best experimental conditions we could achieve. When measuring the distribution function $f(E)$ obtained by sweeping V_{P2}, we have simultaneously corrected for the cross-talk between V_{P1} and V_{P2}, in order to keep a constant injection energy $E_1(V_{\mathrm{P1}})$. Typically, for a step δV_{P2} in QD2, we correct the plunger gate voltage in the QD1 by $\delta V_{\mathrm{P1}} \approx -\delta V_{\mathrm{P2}}/20$.

SUPPLEMENTARY NOTE 3: CONVOLUTION

The transmitted current $I_2(E_2)$ trough the detector QD2 in the sequential tunneling regime reads:

$$I_2(E_2) = \frac{e}{h} \int L_2(E, E_2)\big[f(E) - f_S(E)\big]dE \tag{1}$$

where $f(E)$ is the distribution function to be probe, $f_S(E)$ the equilibrium distribution function at the source of

Spectrum	α_1 (10^{-2} meV/mV)	α_2 (10^{-2} meV/mV)
S1	3.64 ± 0.02	3.97 ± 0.02
S2	3.55 ± 0.03	0.406 ± 0.001
S3	4.80 ± 0.03	6.35 ± 0.03
S4	4.80 ± 0.03	6.35 ± 0.03
S5	4.80 ± 0.03	6.35 ± 0.03
S6	4.79 ± 0.03	4.97 ± 0.02
S7	4.09 ± 0.02	3.97 ± 0.02
S8	1.14 ± 0.02	3.28 ± 0.02

Supplementary Table 2 | Measured lever arms α_i for the resonances used in each QD ($i = 1, 2$) to obtain the spectra discussed in the main text.

the detector, and $L_2(E, E_2)$ is the intrinsic lineshape of the resonance on QD2. A simple model of electrostatic confinement for the electrons in the QD gives $L_2(E, E_2)$ as a Lorentz function centered in E_2.

If the resonance of the detector is narrow enough, namely its intrinsic width $\Gamma_2 \ll k_B T$, the lineshape can be approximated by a normalized delta function $L_2(E, E_2) \sim L_0 \times \delta(E - E_2)$, with $L_0 = \int L_2(E, E_2) dE$ a constant characteristic of the detector. Thus the current $I_2(E_2)$ is directly proportional to $\Delta f(E) = f(E) - f_S(E)$.

In other cases, as for most of the resonance used in our experiment, the measured signal, obtained from $\partial I_2 / \partial V_{\mathrm{P2}}$, is convoluted with the lineshape of QD2 following Eq. 1. As a consequence, the detected Fermi sea is widened, and the effective electronic temperature T_{eff}, that is obtained by fitting a Fermi function, is larger than the 2DEG electronic temperature T, which is measured with a much narrower resonance. This is the case of the Fermi sea in all the spectra presented in the main text. The table 3 summarizes the measured T_{eff}, T and the base temperature T_{ph} for the different spectra. A comparison between T_{eff} and T provides a method to estimate the linewidth Γ_2 of the detector. An equivalent approach can be followed to further characterize the emitter QD.

Spectrum	T_{eff} (mK)	T (mK)	T_{ph} (mK)
S1	40 ± 2	23 ± 1	18.5 ± 0.2
S2	51 ± 2	23 ± 1	18.1 ± 0.1
S3	48 ± 2	30 ± 1	17.9 ± 0.1
S4	67 ± 4	30 ± 1	18.0 ± 0.1
S5	48 ± 2	30 ± 1	17.9 ± 0.1
S6	39 ± 2	27 ± 2	15.9 ± 0.4
S7	59 ± 3	19 ± 1	17.5 ± 0.1
S8	51 ± 2	30 ± 5	15.3 ± 0.1

Supplementary Table 3 | Comparison between the effective electronic temperature T_{eff}, the 2DEG electronic temperature T and the bath temperature T_{ph}.

The convoluted distribution shows also some deviation from an actual Fermi function, mainly by the development of a long tail. In such a case the convoluted distribution function is better described by an *arctangent* function with a characteristic width T_{atn}:

$$f(E) \approx \frac{1}{2} - \frac{1}{\pi} Arctan\left(\frac{E}{k_B T_{atn}}\right) \tag{2}$$

Notice that this functional form is the same as that predicted for a metastable state, which is expected to occur in the relaxation process of a double step distribution function generated by a QPC at low transmission [2]. Thus the effects of the convolution can hamper the experimental investigation of the predicted metastable state [3].

Moreover, the injected QP peak in our experiment is also affected by the convolution at the detector QD. Let us consider the case when the injected QP peak is a Lorentz peak $L_1(E, E_1)$ centered at the injection energy E_1, with amplitude $\mathcal{T}_1$ and width Γ_1. After the convolution with the QD2 lineshape, $L_2(E, E_2)$, the QP peak maintains its

Lorentzian form but with an increased width: $\Gamma_1 + \Gamma_2$, and a reduced height: $\mathcal{T}_1\Gamma_1/(\Gamma_1 + \Gamma_2)$. Importantly, since the characteristics of the detector are the same for all the curves of the same spectra, the exponential decay of the peak height discussed in the main text is not affected by the convolution. As can be seen in Supplementary Table I, the extracted widths of the Lorentzian fits correspond within less than 10 % deviations to the sum $\Gamma_1 + \Gamma_2$.

SUPPLEMENTARY NOTE 4: ELECTROCHEMICAL POTENTIAL SHIFT

An important check in our experiments consists in verifying that the charge current remains conserved in the edge channel, *i.e.* that no charge tunnels from one edge channel to the other. This can be done by calculating the electrochemical potential shift $\Delta\mu$ in the outer edge channel at the detector, given by the integral of the measured distribution function, and comparing it either to the amount of current I_1 stemming from the drain that is transmitted by QD1 (note that in practice we measure the reflected current $1 - I_1$), or to the shift given by the emitter's resonance parameters, namely $\Delta\mu = \frac{\pi}{2}\Gamma_1 T_1$ for a Lorentzian shaped resonance. This is illustrated in Supplementary Fig. 5 for the $L = 480$ nm data shown in main text Fig. 2. We systematically observe a good agreement, indicating that charge current is always conserved in the outer edge channel in our experiments.

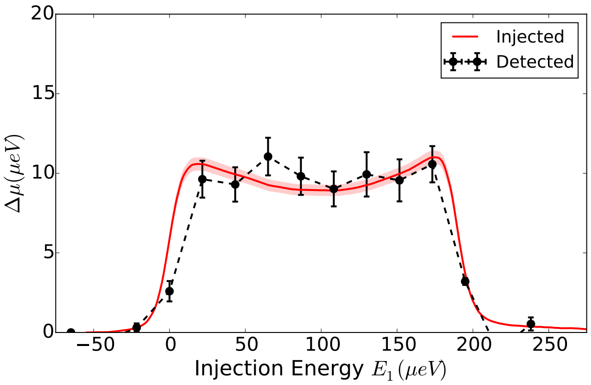

Supplementary Figure 5 | Electrochemical potential shift for $L = 480$ nm. Symbols: electrochemical potential shift $\Delta\mu$ after propagation, obtained by integrating the measured $f(E)$ shown in main text Fig. 2, versus E_1. The error bars correspond to the combined uncertainties on the QDs lever arms and on the normalization of the $f(E)$. Red line: $\Delta\mu$ at the injection, determined from the current flowing through QD1. The red shaded region corresponds to uncertainties on the QDs lever arms. The value of $\Delta\mu$ extracted from the resonance of QD1 is $\frac{\pi}{2}\Gamma_1 T_1 \approx 8.8$ μeV.

SUPPLEMENTARY NOTE 5: ADDITIONAL DATA AND ANALYSIS

We present here the measured distribution functions and their TLL analysis for additional spectra obtained on the 480 nm and 750 nm devices. The spectra (as well as the one shown in the main text) are labelled **Rxx**, where the number **xx** indicates the order in which the data was obtained over the whole measurement run. Spectra R16-R21 where obtained during a first cooldown of the 750 nm device, and spectra R24-R48 during a subsequent cooldown of the same device. Spectra R72-R106 were obtained during a single cooldown of the 480 nm device. The missing numbers correspond to incomplete datasets (*e.g.* due to gate instabilities, or malfunctions of the dilution refrigerator).

Supplementary Fig. 6 shows the spectrum S2, obtained for a slightly broader resonance (labelled resonance B) of the $L = 480$ nm sample, in semi-log scale, along with the Lorentzian fits, the normalized heights of which are shown as black hexagons in main text Fig. 4 (we recall that in the main text, the peak height is normalized by height of the injected quasiparticle peak, given by the transmission of the first quantum dot). The peak positions and FWHM are shown in the inset. Note that in our analysis, we do not remove any background in the distribution functions (regardless of the dataset) before performing the Lorentzian fits. Supplementary Fig. 7 shows the spectrum S4, obtained for a second cooldown (labelled cooldown 2) of the $L = 750$ nm sample, corresponding to the data shown as red and orange circles in main text Fig. 4. We also show in Supplementary Fig. 8 the data and fits appearing in main text Fig. 4, in linear scale. The discrepancy between the data and the fits at small $E_1 \sim 20$ μeV can be accounted for

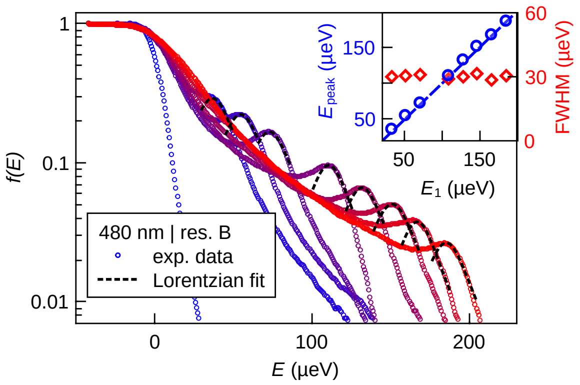

Supplementary Figure 6 | Quasiparticle peak analysis for spectrum S2: $L = 480$ **nm, resonance B.** Measured $f(E)$ in sample #1, plotted in semi-log scale. The inset shows the peak center (blue circles) and the peak width (red diamonds), determined from the Lorentz fit (black dash line) of the peak, as a function of the injection energy E_1.

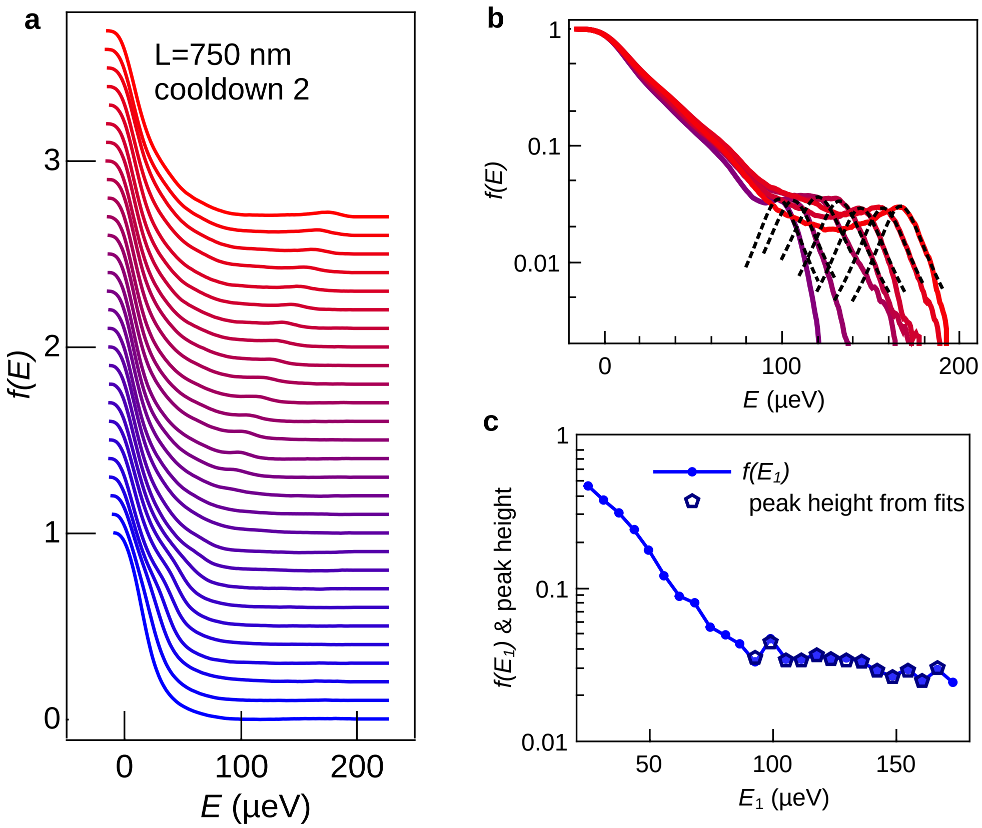

Supplementary Figure 7 | Quasiparticle peak analysis for spectrum S4: $L = 750$ **nm, cooldown 2. a,** Measured $f(E)$. Each curve, offset for clarity, corresponds to an increment of the injection energy $\delta E_1 \approx 6\mu$eV, from $E_1 \approx 6\mu$eV (blue), to $E_1 \approx 173\mu$eV (red). **b,** Measured $f(E)$, plotted in semi-log scale. The black dashed lines are Lorentzian fits of the quasiparticle peak. **c,**, Peak height versus E_1, in semi-log scale. The full blue circles are extracted $f(E_1)$, and the pentagons the peak heights extracted from the Lorentzian fits.

by the fact that at low energy, the quasiparticle peak sits on the tail of the Fermi distribution function corresponding to the Fermi sea of the EC.

Supplementary Fig. 9 shows the spectrum S5, obtained at $L = 750$ nm when the ECs are not separated (see main

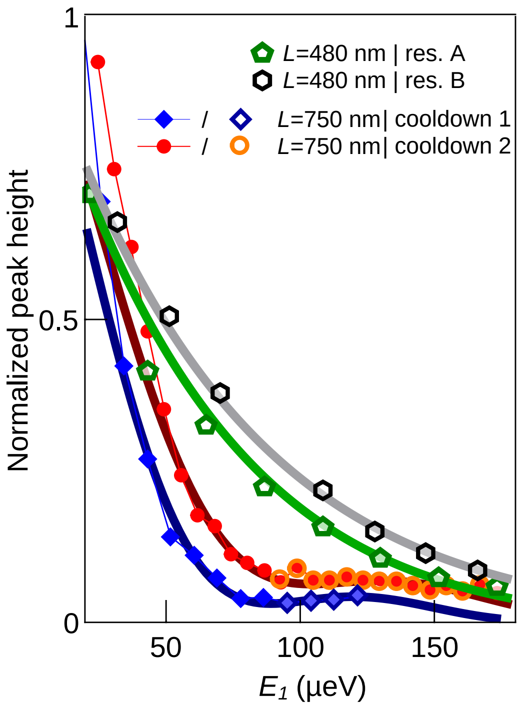

Supplementary Figure 8 | Normalized peak height. Normalized peak height versus E_1 (same data as in main text Fig. 4), in a linear scale. The fitting parameters are reported in Supplementary Table IV.

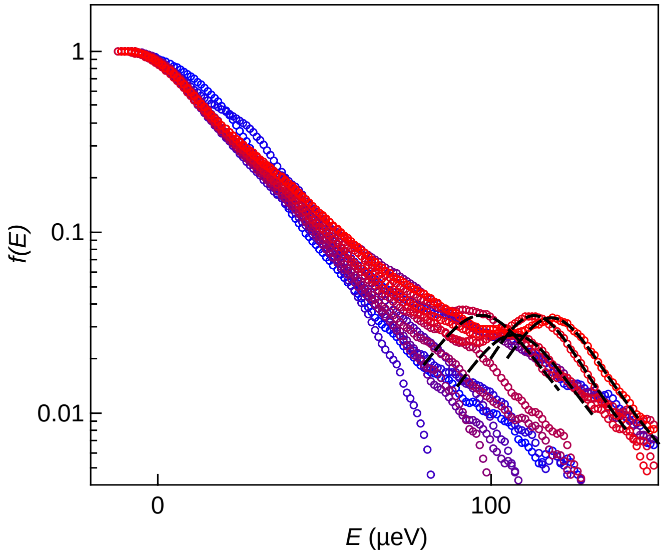

Supplementary Figure 9 | Quasiparticle peak analysis for spectrum S5: $L = 750$ nm with copropagating ECs. Measured $f(E)$ in semi-log scale (symbols), with Lorentzian fits (black dashed lines).

text Fig. 5d), in semi-log scale, among with Lorentzian fits of the high E_1 data where the quasiparticle peak is visible again. The data at $L = 750$ nm when the ECs are separated (see main text Fig. 5e) is shown in semi-log scale in Supplementary Fig. 10. Along with the peak position and FWHM, we show the evolution of the peak height versus E_1. Note that in this case the exponential character of the decay is less clear, as the peak height only decreases by 50 %. We show in Supplementary Fig. 11 the fits of quasiparticle peak height obtained from the above data (750 nm, copropagating and separated ECs), using our refined TLL model. The TLL parameters are summarized in Supplementary Table IV.

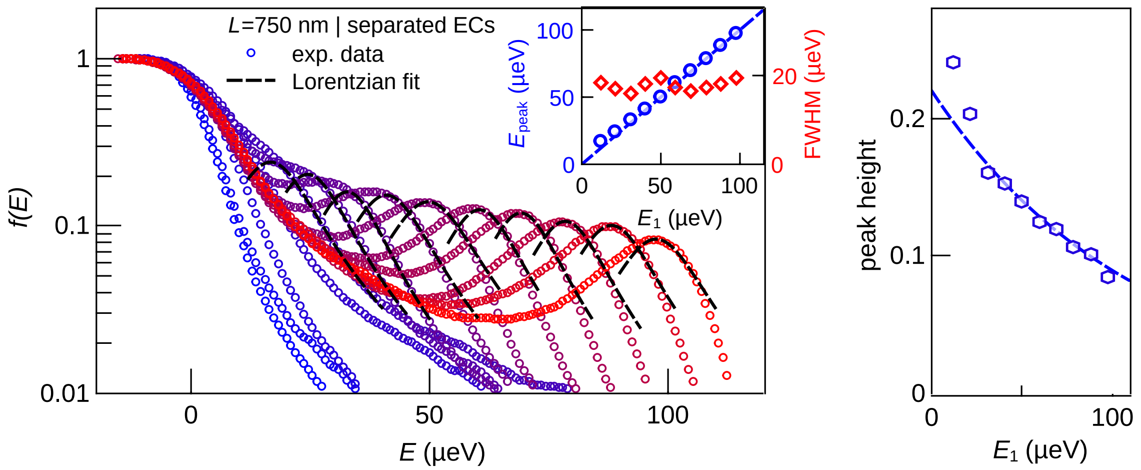

Supplementary Figure 10 | Quasiparticle peak analysis for spectrum S6: $L = 750$ **nm with separated ECs.** Left panel: measured $f(E)$ in semi-log scale (symbols), with Lorentzian fits (black dashed lines). The extracted peak positions and FWHM are shown in the inset. Right panel: absolute peak heights (blue hexagons) extracted from the Lorentzian fits shown in the left panel, plotted in linear scale versus E_1. The blue dashed line is the an exponential fit. The fitting parameters are reported in Supplementary Table IV.

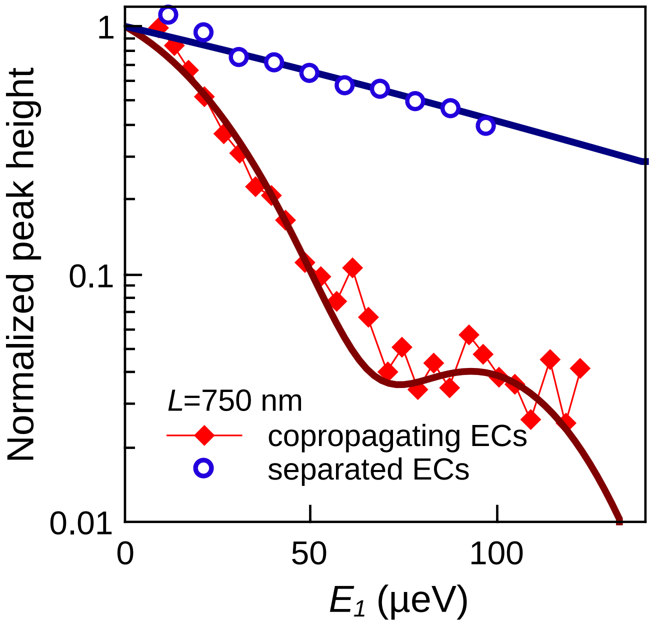

Supplementary Figure 11 | TLL fits of the 750 nm data with copropagating (spectrum S5) /separated (spectrum S6) ECs. Normalized peak height versus E_1. Red diamonds: extracted $f(E_1)$ for copropagating ECs. Blue circles: peak heights from Lorentz fits for separated ECs). The thick lines are fits using the TLL model with dissipation. The fitting parameters are reported in Supplementary Table IV.

Supplementary Figures 12, 13, 14, 15, 16 and 17 show the measured distribution functions as well as the corresponding analysis for the datasets listed in Fig. 6 of the main paper (for both 480 and 750 nm devices), in particular the long path (Fig. 12) and the separated ECs (Fig. 13) configurations of the 480 nm device. The parameters extracted from the TLL analysis are summarized in Supplementary Table IV.

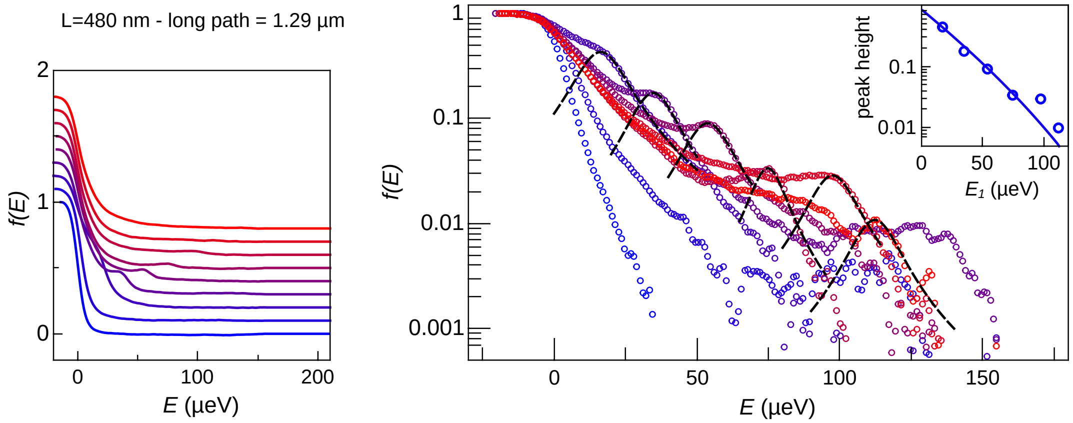

Supplementary Figure 12 | TLL analysis for additional spectrum R76: long path of the 480 nm device. Left Panel: measured $f(E)$. Each curve, offset for clarity, corresponds to an increment $\delta E \approx 19$ μeV of the injection energy. Right panel: measured $f(E)$ in semi-log scale (symbols), with Lorentzian fits (black dashed lines). Inset: peak heights (blue circles) extracted from the Lorentzian fits shown in the left panel, plotted in semi-log scale versus E_1. The blue line is a fit using the TLL model with dissipation. The fitting parameters are reported in Supplementary Table IV.

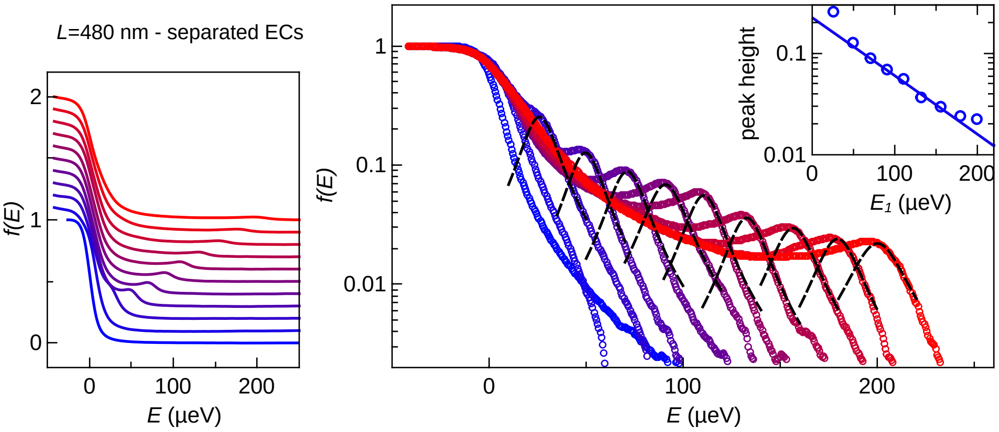

Supplementary Figure 13 | TLL analysis for additional spectrum R84: 480 nm device with separated ECs. Left Panel: measured $f(E)$. Each curve, offset for clarity, corresponds to an increment $\delta E \approx 22$ μeV of the injection energy. Right panel: measured $f(E)$ in semi-log scale (symbols), with Lorentzian fits (black dashed lines). Inset: peak heights (blue circles) extracted from the Lorentzian fits shown in the left panel, plotted in semi-log scale versus E_1. The blue line is a fit using the TLL model with dissipation. The fitting parameters are reported in Supplementary Table IV.

SUPPLEMENTARY NOTE 6: LENGTH DEPENDENCE AND THERMALIZED STATE

At lengths larger than 750 nm, the quasiparticle peak fully vanishes and the measured distribution functions become monotonous. Main text Fig. 6 shows that these present systematic discrepancies with respect to Fermi functions. In particular, fitting the high energy part of the distribution function with a Fermi function systematically leads to an excess of particles at low energy. Measuring equilibrium $f(E)$ (that is, when no additional quasiparticle is emitted by the first quantum dot) at high temperature ($T = 157$ mK, see Fig. 19a) yields much smaller low-energy deviations to a Fermi function (note that in that case the temperature is not a fitting parameter, and is fixed to the actual electron temperature extracted from the characterization of the quantum dots). Note that there is no theoretical prediction for the shape of prethermalized state obtained after the relaxation of finite-energy quasiparticle in addition to a finite temperature Fermi function (such a prediction only exists so far for a biased quantum point contact with extreme

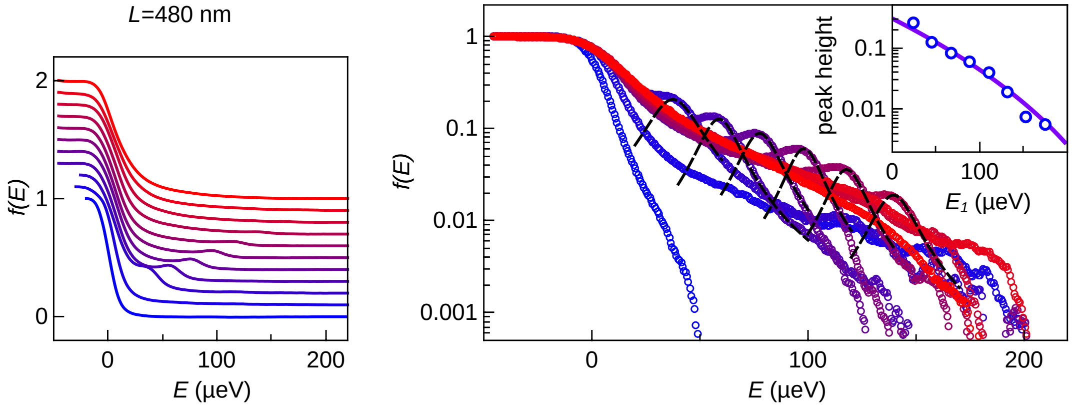

Supplementary Figure 14 | TLL analysis for additional spectrum R99: 480 nm device Left Panel: measured $f(E)$. Each curve, offset for clarity, corresponds to an increment $\delta E \approx 21$ μeV of the injection energy. Right panel: measured $f(E)$ in semi-log scale (symbols), with Lorentzian fits (black dashed lines). Inset: peak heights (blue circles) extracted from the Lorentzian fits shown in the left panel, plotted in semi-log scale versus E_1. The blue line is a fit using the TLL model with dissipation. The fitting parameters are reported in Supplementary Table IV.

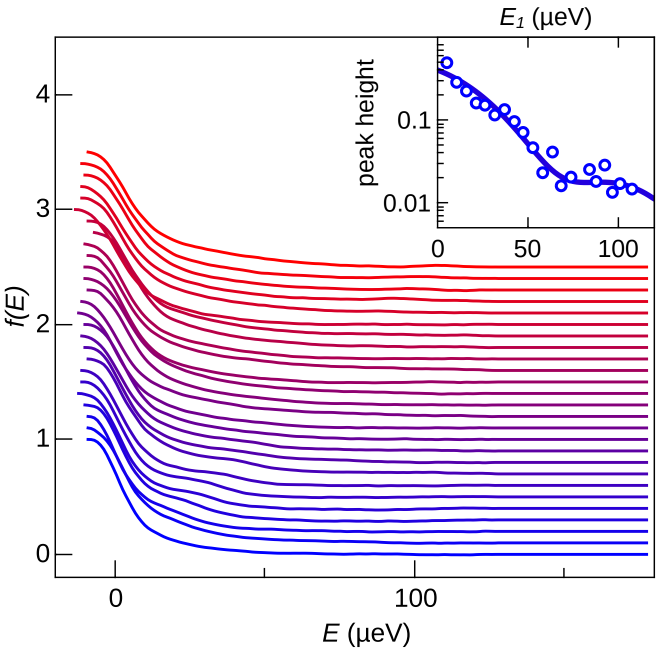

Supplementary Figure 15 | TLL analysis for additional spectrum R16: 750 nm device. Measured $f(E)$. Each curve, offset for clarity, corresponds to an increment $\delta E \approx 5$ μeV of the injection energy. Inset: peak heights $f(E_1)$ (blue circles) plotted in semi-log scale versus E_1. The blue line is a fit using the TLL model with dissipation. The fitting parameters are reported in Supplementary Table IV.

transmission [2, 3]).

SUPPLEMENTARY NOTE 7: COMPARISON WITH PREVIOUS EXPERIMENTS USING DOUBLE STEP DISTRIBUTION FUNCTIONS

It is not straightforward to make direct quantitative comparisons (without using the TLL model) between our experiment and previous experiments, where the initial energy distribution function was a double step function [3, 4].

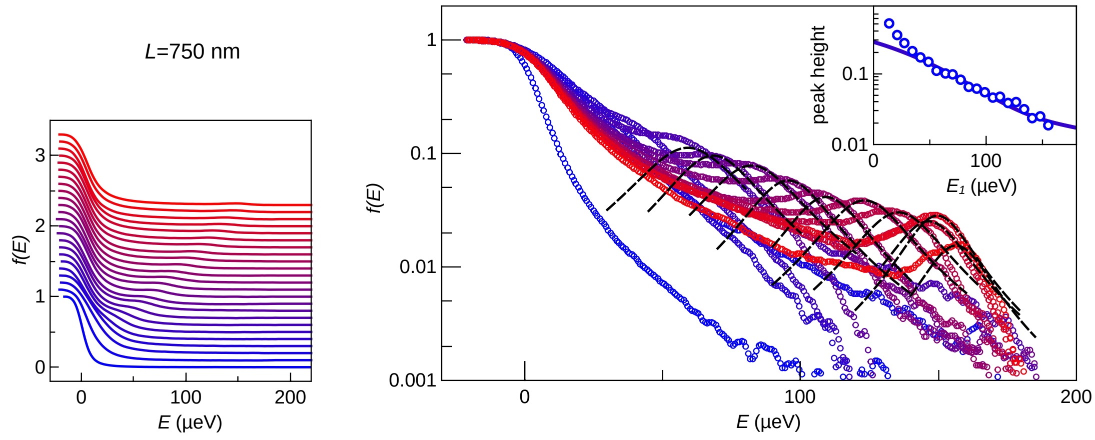

Supplementary Figure 16 | TLL analysis for additional spectrum R40: 750 nm device. Left Panel: measured $f(E)$. Each curve, offset for clarity, corresponds to an increment $\delta E \approx 7$ μeV of the injection energy. Right panel: measured $f(E)$ in semi-log scale (symbols), with Lorentzian fits (black dashed lines). Inset: peak heights (blue circles) extracted from the Lorentzian fits shown in the left panel, plotted in semi-log scale versus E_1. The blue line is a fit using the TLL model with dissipation. The fitting parameters are reported in Supplementary Table IV.

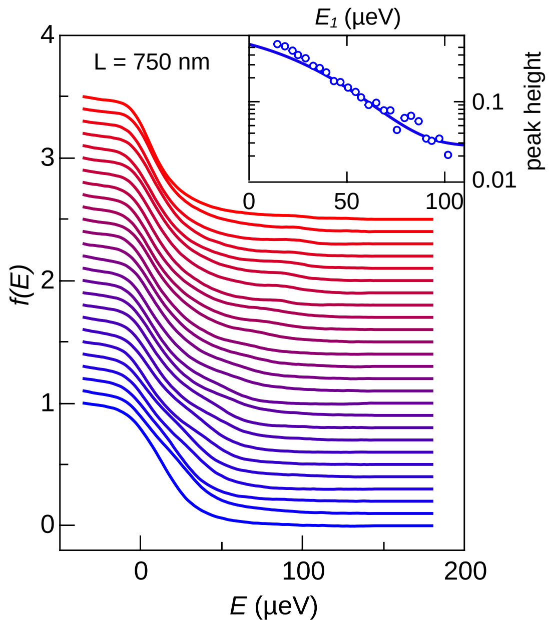

Supplementary Figure 17 | TLL analysis for additional spectrum R48: 750 nm device. Measured $f(E)$. Each curve, offset for clarity, corresponds to an increment $\delta E \approx 4$ μeV of the injection energy. Inset: peak heights $f(E_1)$ (blue circles) plotted in semi-log scale versus E_1. The blue line is a fit using the TLL model with dissipation.The fitting parameters are reported in Supplementary Table IV.

A possible quantifier for relaxation is to compare the relative decrease of the quasiparticle peak in our experiment with the relative decrease in the step widths in refs. [3, 4] (as used explicitly in ref. [3]), as both are adimensional quantities that are shown to decrease with propagation length and energy. By doing so, we obtain the following numbers:

- ref. [3]:

* for $L = 0.5$ μm and $eV_{dc} = 60$ μeV, the step width in the double step distribution function is reduced by 25 %. In comparison, for a similar length and injection energy E_1, the quasiparticle peak amplitude is suppressed by 60 % in our experiment.

* for $L = 0.5$ μm and $eV_{dc} = 180$ μeV, the energy spacing of the double step energy distribution function is reduced

spectrum #	device	length (µm)	v_2 (km/s)	v_ρ (km/s)	v_σ (km/s)	α	θ (×π)	γ_0	E_0 (µeV)	E_γ (µeV)
**S1** – R72	480 nm (res. A)	0.48	48	89	35	1.6	0.16	0.43	403	142
**S2** – R106	480 nm (res. B)	0.48	48	92	42	1.8	0.11	0.43	535	147
R76	480 nm (long path)	1.29	48	94	41	1.8	0.12	0.43	184	55
R84	480 nm (separated ECs)	0.48	48	87	47	1.8	0.04	0.43	712	139
R99	480 nm	0.48	48	103	31	1.8	0.16	0.43	309	164
**S3** – R21	750 nm (cooldown 1)	0.75	38	101	17	2.1	0.17	0.13	85	342
**S4** – R24	750 nm (cooldown 2)	0.75	38	118	18	2.6	0.15	0.12	95	452
**S5** – R20	750 nm	0.75	24	62	13	2.1	0.16	0.13	69	209
**S6** – R39	750 nm (separated ECs)	0.75	38	82	36	2.1	0.07	0.13	278	277
R16	750 nm	0.75	23	62	13	2.25	0.15	0.13	72	209
R40	750 nm	0.75	34	97	25	2.6	0.115	0.13	147	329
R48	750 nm	0.75	29	79	17	2.4	0.14	0.13	95	269

Supplementary Table 4 | TLL fits parameters. Lowest Fermi velocity v_2, charge and dipole plasmon velocities v_ρ and v_σ, Fermi velocities ratio α, effective inter-EC coupling θ, friction coefficient γ_0, revival energy E_0 and exponential decay characteristic energy E_γ extracted from the TLL fits.

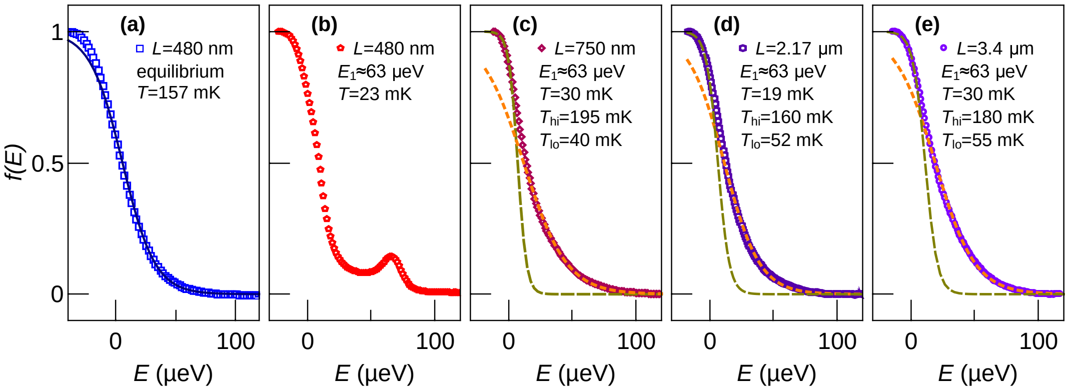

Supplementary Figure 19 | Length dependence. a, measured distribution function at equilibrium, for a base temperature of 157 mK (blue squares). The dark blue line is a Fermi function fit of the data with a fixed temperature of 157 mK. **b-e,** measured $f(E)$ when injecting quasiparticles at $E_1 \approx 63$ μeV, with corresponding Fermi function fits (same data as main text Fig. 5). The temperature T indicates the base electron temperature at which each experiment is performed, while T_{hi} (resp. T_{lo} indicates the temperature of the Fermi function fit at high (resp. low) energy.

by 50 %. For similar length and injection energy, the quasiparticle peak amplitude is suppressed by 92 % in our

experiment.

- ref. [4]:

* for $L = 0.8\ \mu$m and $eV_{dc} = 36\ \mu$eV, the energy spacing of the double step energy distribution function is reduced by 5 %. For similar length and injection energy, the quasiparticle peak amplitude is suppressed by 88 % in our experiment.

These numbers can be put in perspective with the extracted values of the plasmon velocity (the relaxation rate decreases with the velocity): $v^* = 27$ km/s in ref. [3], $v^* = 87$ km/s in ref. [4], and $v^* = 20 - 77$ km/s in our results. Using this quantifier, it thus appears that the quasiparticle peak indeed relaxes "faster", since, for comparable lengths and energies (provided this energy comparison is valid), the relative amplitude suppression is systematically larger than the relative decrease in the double step width. This is emphasized by the fact that the values of the plasmon velocity are similar.

SUPPLEMENTARY NOTE 8: REFINED TLL MODEL INCLUDING DISSIPATION

We consider the edge channels of a quantum Hall bar at filling factor $\nu = 2$, assuming a short range capacitive coupling between them and energy dissipation due to the external environment. According to the experimental observations no electron tunneling occurs between the edges.

The present experiment involves the injection of an electronic wave-packet with Lorentzian profile in energy and its detection after a given length. In order to describe this situation one can proceed as in Refs. [5–7], where the system is divided into a non-interacting injection region, an interacting propagating region and a non-interacting region of detection.

According to this one can describe the interacting region in terms of the conventional Wen's hydrodynamical approach [8] with Hamiltonian density ($\hbar = 1$)

$$\mathcal{H} = \frac{v_1}{4\pi}\left(\partial_x \phi_1\right)^2 + \frac{v_2}{4\pi}\left(\partial_x \phi_2\right)^2 + \frac{u}{2\pi}\partial_x \phi_1 \partial_x \phi_2 \tag{3}$$

where ϕ_1 and ϕ_2 are bosonic fields related to the edge particle density through the condition

$$\rho_{1,2} = \frac{1}{2\pi}\partial_x \varphi_{1,2}, \tag{4}$$

v_1 and v_2 are the bare propagation velocities of the two channels and u the intensity of their coupling. Without loss of generality in the following we will indicate $v_2 = v$ and $v_1 = \alpha v$, with $\alpha > 1$.

Due to the inter-edge interaction the bosonic fields ϕ_1 and ϕ_2 are no longer eigenstates of the Hamiltonian and the system is diagonalized in terms of a charged and a dipole mode, indicated respectively with ϕ_ρ and ϕ_σ with associated eigenvelocities v_ρ and v_σ given by

$$v_{\rho,\sigma} = v f_{\rho,\sigma}\left(\alpha, \theta\right) \tag{5}$$

with

$$f_{\rho,\sigma}\left(\alpha, \theta\right) = \left(\frac{\alpha + 1}{2}\right) \pm \frac{1}{\cos\left(2\theta\right)}\left(\frac{\alpha - 1}{2}\right) \tag{6}$$

and

$$\tan\left(2\theta\right) = \frac{2u}{v\left(\alpha - 1\right)}. \tag{7}$$

It is worth to note that the stability condition of the model imposes the constraint [9]

$$\theta < \frac{1}{2}\arccos\left(\frac{\alpha - 1}{\alpha + 1}\right) < \frac{\pi}{4}. \tag{8}$$

The equations of motion, expressed in Fourier transform with respect to time, become

$$\left(-i\omega + v_\eta \partial_x\right)\tilde{\phi}_\eta(x, \omega) = 0 \qquad \eta = \rho, \sigma. \tag{9}$$

Various experiments [4, 10] suggest a relevant role played by energy dissipation in the transport along quantum Hall edge channels. The simplest way to include this effect in the model is by adding a frequency dependent energy

loss rate $\gamma(\omega)$ (assumed here equal for both channels for sake of simplicity) at the level of the equations of motion in the interacting region (see Eq. (9)). According to this, they read

$$\left[-i\omega+\gamma(\omega)+v_\eta\partial_x\right]\tilde{\phi}_\eta(x,\omega)=0 \qquad \eta=\rho,\sigma. \tag{10}$$

In the following we will focus on a linear dependence [9]

$$\gamma(\omega)=\gamma_0\omega \tag{11}$$

with γ_0 adimensional friction coefficient, even if more involved functional dependences can be considered [10].

The solution of the equations of motion in Eq. (10) is then given by

$$\tilde{\phi}_\eta(x,\omega)=e^{i\frac{\Gamma\omega}{v_\eta}x}\tilde{\phi}_\eta(0,\omega) \qquad \eta=\rho,\sigma \tag{12}$$

with

$$\Gamma=1+i\gamma_0. \tag{13}$$

The initial conditions

$$\begin{aligned}\tilde{\phi}_\rho(0,\omega)&=\cos\theta\tilde{\phi}_1(0,\omega)+\sin\theta\tilde{\phi}_2(0,\omega)\\ \tilde{\phi}_\sigma(0,\omega)&=-\sin\theta\tilde{\phi}_1(0,\omega)+\cos\theta\tilde{\phi}_2(0,\omega)\end{aligned} \tag{14}$$

fix the (possibly frequency dependent) amplitudes at the point of injection $x=0$.

Scattering matrix approach

Proceeding as in Ref. [7] we obtain the edge-magnetoplasmon scattering matrix connecting the incoming (injected) and the outgoing (detected) bosonic fields through the relation

$$\begin{pmatrix}\tilde{\phi}_1(L,\omega)\\ \tilde{\phi}_2(L,\omega)\end{pmatrix}=\hat{S}(L,\omega)\begin{pmatrix}\tilde{\phi}_1(0,\omega)\\ \tilde{\phi}_2(0,\omega)\end{pmatrix}, \tag{15}$$

with

$$\hat{S}=\begin{pmatrix}\cos^2\theta e^{i\omega\Gamma\tau_\rho}+\sin^2\theta e^{i\omega\Gamma\tau_\sigma} & \sin\theta\cos\theta\left(e^{i\omega\Gamma\tau_\rho}-e^{i\omega\Gamma\tau_\sigma}\right)\\ \sin\theta\cos\theta\left(e^{i\omega\Gamma\tau_\rho}-e^{i\omega\Gamma\tau_\sigma}\right) & \sin^2\theta e^{i\omega\Gamma\tau_\rho}+\cos^2\theta e^{i\omega\Gamma\tau_\sigma}\end{pmatrix}. \tag{16}$$

In the above equation we have introduced the short-hand notation $\tau_\alpha=L/v_\alpha$ ($\alpha=\rho,\sigma$) for the times of flight associate to the eigenmodes in the interacting region.

In the following we will focus only on the top left entry of the scattering matrix in Eq. (16), namely

$$t(\omega)=\cos^2\theta e^{i\omega\Gamma\tau_\rho}+\sin^2\theta e^{i\omega\Gamma\tau_\sigma} \tag{17}$$

which represents the amplitude probability for the edge-magnetoplasmon to be transmitted along the first channel (injection channel).

Elastic scattering amplitude

As shown in Ref. [11], assuming a narrow enough (ideally δ-like) injected wave-packet in energy, the evolution of the height of the wave-packet as a function of the injection energy ξ (referred to as E_1 in the main text) is given by

$$\mathcal{V}(\xi)=\frac{|\mathcal{Z}(\xi)|^2}{|\mathcal{Z}(0)|^2} \tag{18}$$

with

$$\mathcal{Z}(\xi)=\int_{-\infty}^{+\infty}d\tau e^{i\xi\tau}\exp\left\{\int_0^{+\infty}\frac{d\omega}{\omega}\left[t(\omega)e^{-i\omega\tau}-1\right]e^{-\omega/\omega_c}\right\} \tag{19}$$

the elastic scattering amplitude, where we introduced a converging factor ω_c (greatest energy scale in the systems).

By replacing the expression for $t(\omega)$ in Eq. (17) one obtains

$$\mathcal{V}(\xi) = e^{-2\frac{\gamma_0}{f_\rho}\frac{\xi}{\xi_0}} \left| {}_1F_1\left[p_+, 1; -\gamma_0 \frac{\xi}{\xi_0}\left(\frac{1}{f_\sigma} - \frac{1}{f_\rho}\right) + i\frac{\xi}{\xi_0}\left(\frac{1}{f_\sigma} - \frac{1}{f_\rho}\right)\right]\right|^2 \Theta(\xi) \tag{20}$$

with

$$\xi_0 = \frac{\hbar v}{L} \tag{21}$$

and where ${}_1F_1[a, b; z]$ is the Kummer confluent hypergeometric function. This quantity depends on four free parameters, namely α, θ, γ_0 and ξ_0, which need to be fixed in order to fit the experimental data.

It is worth noticing that in the "strongly interacting" limit ($\theta = \pi/4$) and in absence of dissipation ($\gamma_0 = 0$) the above expression reduces to

$$\mathcal{V}_{strong}(\xi) = \left| J_0\left(\frac{\xi}{\xi^*}\right)\right|^2 \Theta(\xi) \tag{22}$$

with J_0 zero-th order Bessel function and where we introduced the parameter

$$\xi^* = 2\left(\frac{L}{\hbar v_\sigma} - \frac{L}{\hbar v_\rho}\right)^{-1}. \tag{23}$$

This expression is the one considered in Ref. [6].

Notice that the value of ξ_0 sets the position of the revival. Moreover, from this value and from the knowledge of the length L of the interacting region reported in the experimental paper it is possible to extract the value of the bare velocity v.

[1] C. Rössler, S. Burkhard, T. Krähenmann, M. Röösli, P. Märki, J. Basset, T. Ihn, K. Ensslin, C. Reichl, and W. Wegscheider, Physical Review B **90**, 081302 (2014).

[2] I. P. Levkivskyi and E. V. Sukhorukov, Physical Review B **85**, 075309 (2012).

[3] K. Itoh, R. Nakazawa, T. Ota, M. Hashisaka, K. Muraki, and T. Fujisawa, Physical Review Letters **120**, 197701 (2018).

[4] H. le Sueur, C. Altimiras, U. Gennser, A. Cavanna, D. Mailly, and F. Pierre, Physical Review Letters **105**, 056803 (2010).

[5] P. Degiovanni, C. Grenier, G. Fève, C. Altimiras, H. le Sueur, and F. Pierre, Physical Review B **81**, 121302 (2010).

[6] D. Ferraro, B. Roussel, C. Cabart, E. Thibierge, G. Fève, C. Grenier, and P. Degiovanni, Physical Review Letters **113**, 166403 (2014).

[7] D. Ferraro and E. Sukhorukov, SciPost Physics **3**, 014 (2017).

[8] X.-G. Wen, Adv. Phys. **44**, 405 (1995).

[9] A. Braggio, D. Ferraro, M. Carrega, N. Magnoli, and M. Sassetti, New J. Phys. **14**, 093032 (2012).

[10] E. Bocquillon, V. Freulon, J.-M. Berroir, P. Degiovanni, B. Plaçais, A. Cavanna, Y. Jin, and G. Fève, Nature Communications **4**, 1839 (2013).

[11] P. Degiovanni, C. Grenier, and G. Fève, Physical Review B **80**, 241307 (2009).
